# Giant Electroresistance in Edge Metal-Insulator-Metal Tunnel Junctions Induced by Ferroelectric Fringe Fields

**DOI:** 10.1038/srep30646

**Published:** 2016-08-01

**Authors:** Sungchul Jung, Youngeun Jeon, Hanbyul Jin, Jung-Yong Lee, Jae-Hyeon Ko, Nam Kim, Daejin Eom, Kibog Park

**Affiliations:** 1 Department of Physics, Ulsan National Institute of Science and Technology (UNIST), Ulsan 44919, Republic of Korea; 2 School of Electrical and Computer Engineering, Ulsan National Institute of Science and Technology (UNIST), Ulsan 44919, Republic of Korea; 3 Department of Physics, Hallym University, Chuncheon Gangwondo 24252, Republic of Korea; 4 Korea Research Institute of Standards and Science, Daejeon 34113, Republic of Korea

## Abstract

An enormous amount of research activities has been devoted to developing new types of non-volatile memory devices as the potential replacements of current flash memory devices. Theoretical device modeling was performed to demonstrate that a huge change of tunnel resistance in an Edge Metal-Insulator-Metal (EMIM) junction of metal crossbar structure can be induced by the modulation of electric fringe field, associated with the polarization reversal of an underlying ferroelectric layer. It is demonstrated that single three-terminal EMIM/Ferroelectric structure could form an active memory cell without any additional selection devices. This new structure can open up a way of fabricating all-thin-film-based, high-density, high-speed, and low-power non-volatile memory devices that are stackable to realize 3D memory architecture.

Ferroelectric materials have been studied quite extensively for non-volatile memory applications relying on their spontaneous polarization^1^,^2^. It has been shown that the energy band profile across the ferroelectric layer of a metal/ferroelectric/metal tunnel junction changes greatly depending on polarization direction and the resulting switching ratio of the tunnel current becomes large enough to be used as a memory device^3^,^4^,^5^. Ferroelectric field effect transistors have also been studied much for memory applications^6^,^7^,^8^,^9^,^10^,^11^. However, the difficulty of growing very thin (several nm thick) high-quality ferroelectric films has hampered fabricating memory devices that show the operational performances predicted theoretically and are reliable enough for actual commercialization. In this study, we performed theoretical device modeling to calculate the tunnel current in an Edge Metal-Insulator-Metal (EMIM) junction formed over the sidewall of a metal electrode. The tunnel current of EMIM junction was found to be strongly influenced by the electric fringe field^12^ originating from an underlying ferroelectric layer. Finite-element electrostatic modeling was used to find the energy barrier profile through the insulating layer of EMIM junction and the tunnel current through the energy barrier was calculated based on transfer matrix method^13^,^14^,^15^. The switching ratio of tunnel current between two opposite polarization directions in the ferroelectric layer was found to be very large (~10^13^), which enables the clear non-destructive read-out of the ferroelectric memory device composed of a stack of EMIM junction and ferroelectric layer. We also demonstrate that the EMIM/Ferroelectric stack can form an active memory cell with no need of any additional selection devices (transistors or diodes) to be selected unambiguously in a cross-bar type of memory cell arrangement. This would make it possible to construct a high density, high speed, low power, and stackable memory device based all on thin films.

## Results

### Device structure of EMIM junction and electron energy band profile of its tunnel insulator obtained by performing finite-element electrostatic modeling

**Figure 1:**
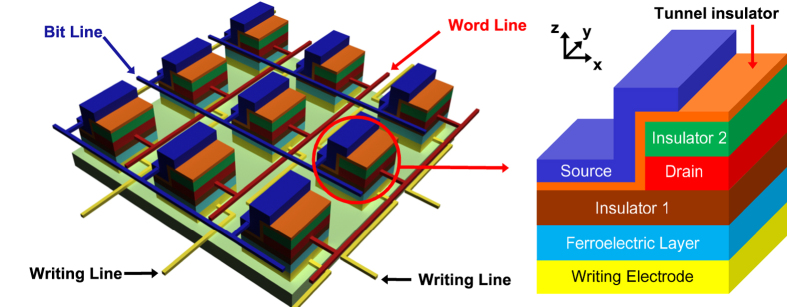
Schematic view of EMIM/Ferroelectric memory cell array. The zoom-in shows the detailed layer structure of each memory cell. The source electrodes of memory cells are connected to the bit lines, the drain electrodes to the word lines, and the writing electrodes to the writing lines.

**Figure 2:**
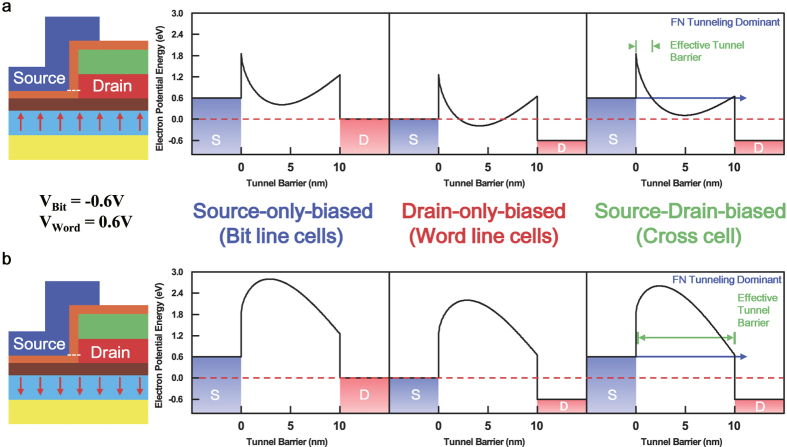
Electron energy band profiles across tunnel insulator. (**a**) Polarization up-state (Written) and (**b**) Polarization down-state (Erased). The energy band profiles are taken along the white dotted lines in the device structure schematic views. The bit line cells are source-only biased, the word line cells are drain-only biased, and the cross cells are both source and drain biased. The red dotted line indicates the Fermi level of source or drain electrode at zero bias voltage. The Fowler-Nordheim (FN) tunneling becomes dominant in cross cells.

The 3-dimensional schematic view of an array of EMIM/Ferroelectric memory cells is shown in Fig. 1. The EMIM junction is formed by covering one sidewall of the drain electrode with the thin tunnel insulator and the source electrode so that the tunnel junction is rotated by 90° in comparison with the conventional vertical one (Zoom-in of Fig. 1). The electron energy band profiles across the tunnel barrier for the two different polarization directions in the underlying ferroelectric layer are depicted in Fig. 2a,b respectively. The electron energy band profile was obtained by performing finite-element electrostatic modeling with the commercial package FlexPDE^16^. In our device modeling, the material for each layer was assumed as follows: Pb_1.1_Zr_0.35_Ti_0.65_O_3_ for the ferroelectric layer (400 nm) with 25 μC/cm^2^ remnant polarization^17^,^18^, SiO_2_ for the blocking insulator 1 (8 nm) and the blocking insulator 2 (20 nm), SiC for the tunnel insulator (10 nm), and Pt for the source, drain, and writing electrodes (30 nm each). The thicknesses, dielectric constants, and electron affinities of ferroelectric and insulator layers are listed in Table 1. The workfunction of Pt was selected to be 5.1 eV^19^,^20^. As shown in Fig. 2, the calculated energy band profile is drastically different between polarization up-state (Fig. 2a) and down-state (Fig. 2b) of the ferroelectric layer. In case of polarization up-state, positive polarization charges are induced on the surface of the ferroelectric layer and these positive polarization charges lead to the valley-shaped energy band profile in the tunnel insulator. For polarization down-state, negative polarization charges are induced on the surface of the ferroelectric layer, which arouse the ridge-shaped energy band profile in the tunnel insulator. The change of the energy band profile in the tunnel insulator mostly occurs near the bottom close to the underlying ferroelectric layer and it diminishes somewhat quickly as going up away from the ferroelectric layer (Supplementary Fig. 1S). Here, we note the functionalities of the two blocking insulators labeled in the zoom-in of Fig. 1. The blocking insulator 1 formed on the ferroelectric layer reduces the screening of the fringe electric field coming from the ferroelectric layer by the source and drain electrodes, and also blocks the leakage current through the ferroelectric film. The blocking insulator 2 forces the electrical current between the source and drain electrodes to flow only through the EMIM junction by preventing any electrical current flow into the top horizontal plane of the drain electrode.

### Calculation of tunnel current through tunnel insulator of EMIM junction

Once the energy band profile in the tunnel insulator is obtained, the tunnel current through the tunnel insulator is calculated. First, the transfer matrix method^13^,^14^,^15^ is adopted to solve the 1-dimensional effective mass Schrödinger equation^21^ given as


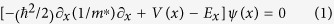


where m^*^ is the effective mass of electron, 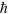 the reduced Planck’s constant, *V*(*x*) the potential energy, and *E*_*x*_ the electron energy along the direction going across the tunnel insulator. We divide the energy band profile into small rectangular segments each of which has a width of *dx*. The effective mass can vary from segment to segment but it is assumed to be constant in each segment. Then, the wave function *ψ*_*j*_(*x*) in the *j*-th segment satisfies the conventional 1-dimensional Schrödinger equation


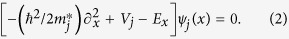


here, 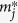 and *V*_*j*_ are the effective mass of electron and the constant potential energy respectively in the *j*-th segment. The general solution for the 1-dimensional Schrödinger equation in the *j*-th segment is obtained to be


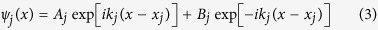


with arbitrary coefficients *A*_*j*_ and *B*_*j*_ where 
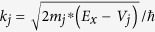
 and *x*_*j*_ is the left edge position of the *j*-th segment. From the proper boundary conditions between two neighboring segments (The wave functions and their first derivatives are continuous.), the following relation connecting the wave functions in the two segments with the transfer matrix *T*_*j+*1*, j*_ can be derived.


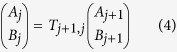



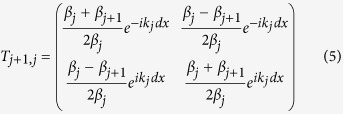


where 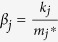


With the source and drain electrodes included, we need *N*+2 wave functions and *N*+1 transfer matrices if dividing the energy band profile of the tunnel barrier into *N* segments (Supplementary Fig. 2S). By multiplying the *N*+1 transfer matrices, we can get the final transfer matrix connecting the wave functions in the source and drain electrodes. As shown below, we can calculate the tunneling probability of electron through the given energy band profile from the final transfer matrix.


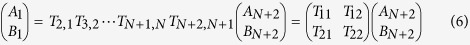


Since electrons are supplied from the source electrode with a positive drain voltage, there will be no electrons moving leftward in the drain electrode and consequently *B*_*N*+2_=0. Then, the electron tunneling probability is calculated to be 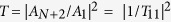. By adopting the generalized tunneling formula proposed by Simmons^22^, the tunnel current density (tunnel current per unit area) can be expressed as


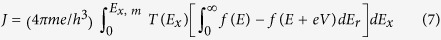


where *E* = *E*_*x*_ + *E*_*r*_, *E*_*x*_ is the electron energy in the *x*-direction and *E*_*r*_ is the electron energy in the plane perpendicular to the *x*-direction. *V* is the applied drain voltage, *m* the free electron mass, *e* the magnitude of electron charge, and *h* Planck’s constant. *T*(*E*_*x*_) is the tunneling probability depending on *E*_*x*_. *E*_*x, m*_ is the upper limit of the integral for *E*_*x*_. By using mensuration by parts, the tunnel current density can be calculated numerically as


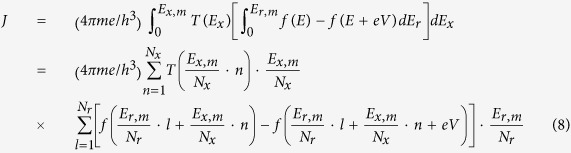


where *N*_*x*_ and *N*_*r*_ are the number of parts for *E*_*x*_ and *E*_*r*_ respectively. *E*_*x, m*_ and *E*_*r, m*_ are the upper limits of the integrals for *E*_*x*_ and *E*_*r*_ which are chosen properly to include the tunneling process only and also for convenience in calculation (Details in Method). As pointed out previously, the energy band profile across the tunnel insulator changes as going upward away from the underlying ferroelectric layer. Hence, the tunnel current densities at different heights along the vertical direction in the tunnel insulator (Supplementary Fig. 1S(a)) were integrated to obtain the total tunnel current. The integration of the tunnel current densities at different heights was done by dividing the tunnel insulator into small segments along the vertical direction and by using mensuration by parts. Here, it is noted that the tunnel current density at each height has a unit of A/m^2^ and hence the integrated total tunnel current has a unit of A/m. The channel width of EMIM junction is not specified by considering the translational symmetry along the channel width direction (y-direction in Fig. 1). Thus, the total tunnel current through the EMIM junction is calculated for a unit channel width.

**Figure 3:**
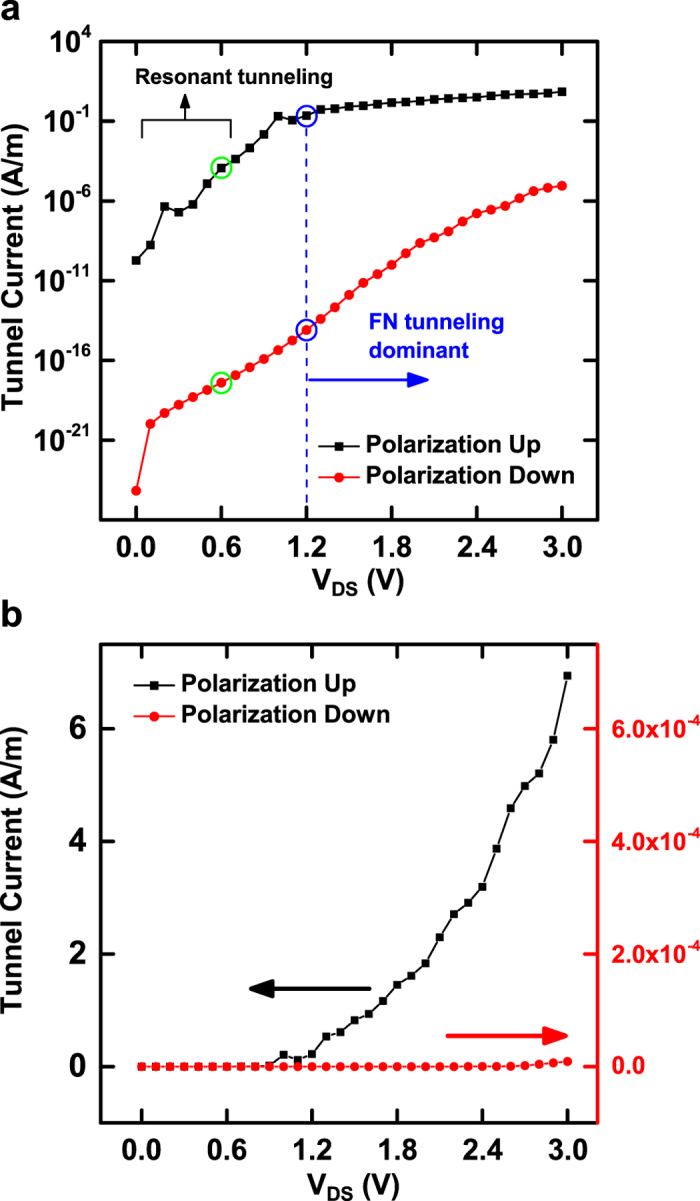
Calculated tunnel current through EMIM junction for a range of source-drain voltage. (**a**) Semi-log scale plot and (**b**) linear scale plot. The linear scale plot has two different y scales, one for polarization up-state (left black) and the other for polarization down-state (right red). It is noted that the tunnel current through the EMIM junction is calculated for a unit channel width (y-direction in Fig. 1) and hence it has the unit of A/m.

The calculated total tunnel currents for polarization up- and down-state are shown in Fig. 3. As shown in the figure, the tunnel current of polarization up-state is much larger than that of polarization down-state. This large difference in tunnel current is expectable based on the energy band profile of tunnel insulator shown in Fig. 2. The valley-shape energy band profile of polarization up-state makes its effective tunnel barrier much thinner than that of polarization down-state (Ridge-shape). A very large ratio (~10^13^) of total tunnel current between polarization up- and down-state occurs at the source-drain voltage of ~1.2 V where the tunnel current appears to turn on in the linear scale plot (Fig. 3b). As described in Supplementary Fig. 1S, the energy band profile of tunnel insulator for polarization up- and down-state become very similar to each other as the height (z-direction) increases. Accordingly, the tunnel current densities at the large-height regions of the tunnel insulator would also be very similar for polarization up- and down-state. In addition, the tunnel current densities at the large-height regions are quite small due to the low tunnel probability through the 10 nm thick tunnel insulator. For polarization up-state, the total tunnel current will be dominated by the contributions from the near-bottom regions close to the ferroelectric layer (Valley-shape). In contrast, for polarization down-state, the tunnel current densities in the near-bottom regions are even smaller (Ridge-shape) than those in the large-height regions, leading to the total tunnel current way smaller than that of polarization up-state. The ratio of total tunnel current is over 10^5^ for the source-drain voltage range of 0.0~3.0 V. With this large ratio of total tunnel current, it is possible to clearly distinguish the polarization direction of the underlying ferroelectric layer. It is noted here that the oscillation in the total tunnel current of polarization up-state, denoted in Fig. 3a, is due to the resonant tunneling through the double tunnel barrier^23^,^24^. As shown in Fig. 2a, double energy barriers can form for some electron energies in all three bias configurations (source-only biased, drain-only biased, and source-drain biased). This resonant tunneling phenomenon for polarization up-state is manifested mainly at small source-drain voltages. Then, it decreases as the source-drain voltage increases because the Fowler-Nordheim tunneling^25^ becomes overwhelming for large source-drain voltages. The Fowler-Nordheim tunneling starts at the source-drain voltage of ~1.2 V around which the tunnel current turns on as described previously.

### Non-destructive read-out and self-selective ferroelectric memory device based on EMIM/Ferroelectric stack

**Figure 4:**
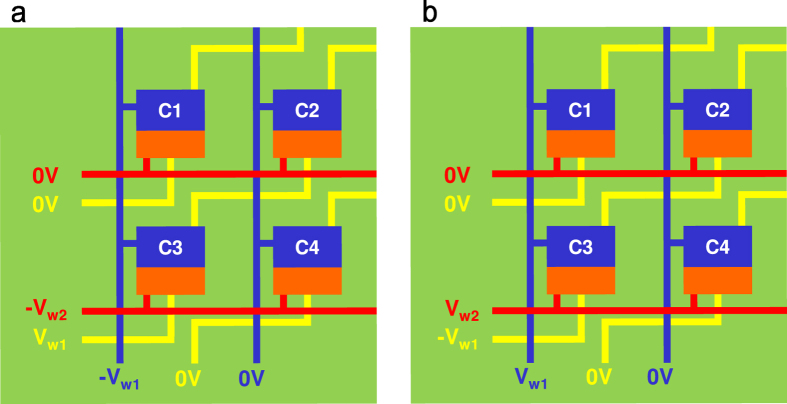
Writing and erasing mechanisms in EMIM/Ferroelectric memory cell array. Only the cross cell denoted as (C3) can be (**a**) written or (**b**) erased by applied voltages. The other cells are not affected since the applied field is smaller than the coercive field of ferroelectric layer.

Based on the calculated tunnel current characteristics of EMIM junction on ferroelectric layer, we can set up the reading and writing mechanisms for EMIM/Ferroelectric memory cell array with no need of selection device (transistor or diode). The source electrodes are connected to the bit lines, the drain electrodes to the word lines, and the writing electrodes to the writing lines as represented in Fig. 1. Here, the polarization up-state is treated as a written-state and the polarization down-state as an erased-state. The written-state can be obtained by applying a positive voltage (V_w1_) on the writing line, the same magnitude of negative voltage (−V_w1_) on the bit line, and a smaller magnitude of negative voltage (−V_w2_) on the word line. In order to write only the cross cell where the three lines are crossed, the applied voltages are selected such that V_w1_ produces the electric field smaller than the coercive field in ferroelectric layer but V_w1_ + V_w2_ and 2V_w1_ induce the electric fields larger than the coercive field (Details in Method). Figure 4a visualizes the writing mechanism described above. When bias voltages are applied on the word (horizontal red), bit (vertical blue), and writing (diagonal yellow) lines as depicted in the figure, the C1 (Cell 1) and C4 (Cell 4) will have only the negative voltage (−V_w1_ or −V_w2_) on either source or drain electrode. For the C2 (Cell 2), only the positive voltage (V_w1_) will be applied on its writing electrode. Therefore, there will be no change in polarization direction for the C1, C2, and C4 since the applied electric fields across their ferroelectric layers are less than the coercive field. Meanwhile, the C3 (Cell 3) will have the positive voltage (V_w1_) on the writing electrode and the negative voltages (−V_w1_ and −V_w2_) on source and drain electrodes. Then, the applied electric field across the ferroelectric layer of the C3 is larger than the coercive field, enforcing the cell to be in polarization up-state (written-state). The erased-state can be obtained in an opposite way, meaning that a negative voltage (−V_w1_) is applied on the writing line, the same magnitude of positive voltage (V_w1_) on the bit line, and a smaller magnitude of positive voltage (V_w2_) on the word line as represented in Fig. 4b. Similarly to the written-state, only the C3 will be enforced to be this time in polarization down-state (erased-state) while the C1, C2, and C4 will keep their polarization states. With these careful choices of the applied voltages, it can be assured that only the cross cell is written or erased without changing the memory states of other cells, including the ones with only ±V_w1_ or ±V_w2_ applied.

For reading out the memory state of a cross cell, an appropriate set of voltages are applied on the corresponding bit and word lines. The important factor in determining the read-out voltages is the turn-on voltage of Fowler-Nordheim tunneling through the tunnel barrier of EMIM structure. As shown in Fig. 2, if applying a positive voltage of 0.6 V on the word line and a negative voltage of −0.6 V on the bit line, only the cross cell is biased with the full turn-on voltage. The cells which are on either bit or word line will be biased just with the half of turn-on voltage. According to the calculated total tunnel current (Fig. 3), the tunnel current of written-state (Blue circle on black curve) is much higher (~10^13^ times) than that of erased-state (Blue circle on red curve) at the turn-on voltage. Also, the written-state tunnel current at the turn-on voltage (Blue circle on black curve) is much higher (~10^3^ times) than the written-state tunnel current at the half of the turn-on voltage (Green circle on black curve). Therefore, the tunnel current of cross cell in written-state is significantly larger than any other cells. Then, the read-out current measured between the corresponding bit and word lines will be completely dominated by the cross cell current. In result, the written-state of the cross cell can be determined unambiguously without any selection device even if there are written memory cells in the word or bit line. In case that the cross cell is in erased-state, the read-out current will be much smaller in comparison with the cross cell being in written-state. It is because the tunnel currents of both erased cross cell (Blue circle on red curve) and other written cells in the word or bit line (Green circle on black curve) are a lot smaller than the tunnel current of written cross cell (Blue circle on black curve). Hence, the erased-state of the cross cell can also be read out unambiguously with no need of selection device.

## Discussion

In summary, we demonstrated with theoretical device modeling that the tunnel current in an EMIM junction of metal crossbar structure can vary drastically depending on the polarization direction of an underlying ferroelectric layer. The electron energy band profile of tunnel barrier was obtained by performing finite element electrostatic modeling and the transfer matrix method was used for calculating the tunnel current density. Our device modeling suggests that the EMIM/Ferroelectric stack can be used to build a cross-bar type of memory cell array which doesn’t require any additional selection devices. With this proposed structure, it is possible to fabricate all-thin-film-based, high-density, high-speed, low-power, and stackable non-volatile memory devices.

## Methods

### Determination of upper limits of integral

In order to calculate the two integrals in Eq. 8 numerically with mensuration by parts, we need to specify the upper limit of each integral. Since only the tunneling process is considered, *E*_*x*_ should NOT go beyond the energy barrier of tunnel insulator. In case of polarization up-state, the energy band profile of tunnel insulator has the valley-shape (Fig. 2a). Therefore, the upper limit (*E*_*x, m*_) is determined by adding the Fermi energy of source metal (*E*_*F, source*_) and the energy difference between the workfunction of source metal (Φ_*source*_) and the electron affinity of tunnel insulator (*χ*_*tunnel*_) as depicted in Supplementary Fig. 2S.


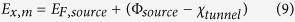


On the other hand, the energy band profile of polarization down-state has the ridge-shape (Fig. 2b). Hence, *E*_*x, m*_ is supposed to be given higher than the value in Eq. 9. However, the same *E*_*x, m*_ as in Eq. 9 was used even for polarization down-state in the calculation. It is because the electron density in the source metal decreases quickly above the Fermi energy by following the Fermi-Dirac distribution^26^. At room temperature (300 K), the Fermi-Dirac distribution decays to 0.001 when the electron energy is 0.3 eV higher than the Fermi energy. Since 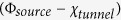 is ~1.25 eV in our case, there will be almost no difference in the calculated tunnel current for any *E*_*x, m*_ higher than the value in Eq. 9. The upper limit of electron energy in the plane perpendicular to the x-direction (*E*_*r, m*_) is also needed to be specified, which is infinity in the Simmons formula as shown in Eq. 7. As mentioned just before, the energies of the electrons contributing to the tunnel current will go beyond the Fermi energy of the source metal only slightly. If *E*_*r, m*_ is chosen to be 0.3 eV higher than the source metal Fermi energy as in Eq. 10 below, all the tunneling electrons are expected to be included in the calculation.


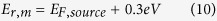


### Determination of writing voltages (V_w1_, V_w2_)

The external field larger than the coercive field should be applied to reverse the polarization direction in the ferroelectric layer. By considering the structure of our EMIM junction (Fig. 1), the layer stack under the drain electrode can be considered as the series connection of two capacitors (*C*_1_: Capacitance of ferroelectric layer, *C*_2_: Capacitance of SiO_2_ layer). When a voltage (*V*_*appl*_) is applied across the stack, the voltage across each layer (*V*_1_: Ferroelectric layer, *V*_2_: SiO_2_ layer) can be obtained by the following relations.


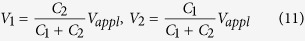


In case of the source electrode, the underlying layer stack is the series connection of three capacitors (*C*_1_: Capacitance of ferroelectric layer, *C*_2_: Capacitance of SiO_2_ layer, *C*_3_: Capacitance of SiC layer). The voltage across each of these three layers becomes the following.


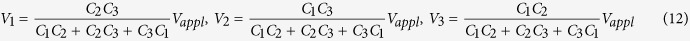


With the voltage division based on the simple parallel capacitor model above, it is confirmed from finite element electrostatic modeling that almost uniform external electric fields can be induced throughout the ferroelectric layer. As can be noted in Eqs. 11 and 12, the source electrode (V_w1_) needs an applied voltage larger than the drain electrode (V_w2_) with the common voltage applied on the bottom writing electrode in order to flip the polarization in the entire region of ferroelectric layer uniformly.

## Additional Information

**How to cite this article**: Jung, S. *et al*. Giant Electroresistance in Edge Metal-Insulator-Metal Tunnel Junctions Induced by Ferroelectric Fringe Fields. *Sci. Rep.* **6**, 30646; doi: 10.1038/srep30646 (2016).

## Supplementary Material

Supplementary Information

## Figures and Tables

**Table 1:** Thickness, dielectric constant, and electron affinity of underlying ferroelectric layer, insulator 1 and 2, and tunnel insulator.

	Thickness (nm)	Dielectric constant	Electron affinity (eV)
Ferroelectric layer (Pb_1.1_Zr_0.35_Ti_0.65_O_3_)	400	350^a^	3.5^b^
Insulator 1 (SiO_2_)	8	3.9^c^	0.9^c^
Insulator 2 (SiO_2_)	20	3.9^c^	0.9^c^
Tunnel insulator (SiC)	10	4.6^d^	3.85^e^

The dielectric constants and electron affinities are taken from ^a^ref. 18, ^b^ref. 2, ^c^ref. 27, ^d^ref. 28, and ^e^ref. 29.
