# Supplementary material for: Giant Electroresistance in Edge Metal-Insulator-Metal Tunnel Junctions Induced by Ferroelectric Fringe Fields
Source: Sci Rep. 2016 Aug 1;6:30646. doi: 10.1038/srep30646 (PMC4967890; doi:10.1038/srep30646)
Supplement: Supplementary Information [file srep30646-s1.doc]

**Giant Electroresistance in Edge Metal-Insulator-Metal Tunnel Junctions Induced by Ferroelectric Fringe Fields**

Sungchul Jung^1^†, Youngeun Jeon ^2^†, Hanbyul Jin^2^, Jung-Yong Lee^1^, Jae-Hyeon Ko^3^, Nam Kim^4^, Daejin Eom^4^, and Kibog Park^1,2^*

^1^Department of Physics, Ulsan National Institute of Science and Technology (UNIST), Ulsan 44919, Republic of Korea

^2^School of Electrical and Computer Engineering, Ulsan National Institute of Science and Technology (UNIST), Ulsan 44919, Republic of Korea

^3^Department of Physics, Hallym University, Chuncheon Gangwondo 24252, Republic of Korea

^4^Korea Research Institute of Standards and Science, Daejeon 34113, Republic of Korea

**Corresponding Author**

Correspondence and requests for materials should be addressed to Kibog Park* (Email: [kibogpark@unist.ac.kr](mailto:kibogpark@unist.ac.kr))

**Equal Contribution**

†These authors contributed equally to this work.


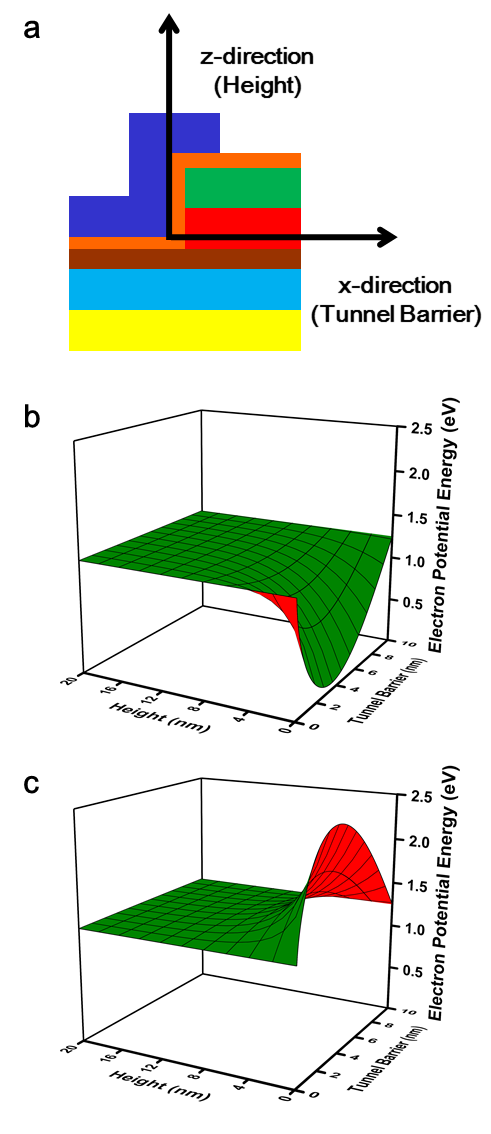


Supplementary Figure 1S.

(**a**) Definition of coordinates for EMIM junction. 2-dimensional electrostatic potential energy profiles for electron of tunnel insulator with zero source-drain bias for (**b**) polarization up-state and (**c**) polarization down-state


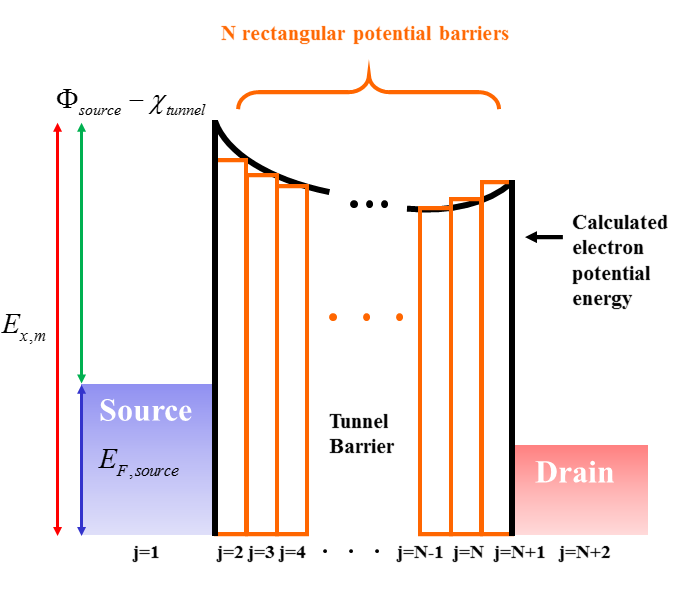


Supplementary Figure 2S.

Illustration demonstrating the division of electron energy band profile of tunnel barrier into N rectangular potential barriers (from j=2 to j=N+1)


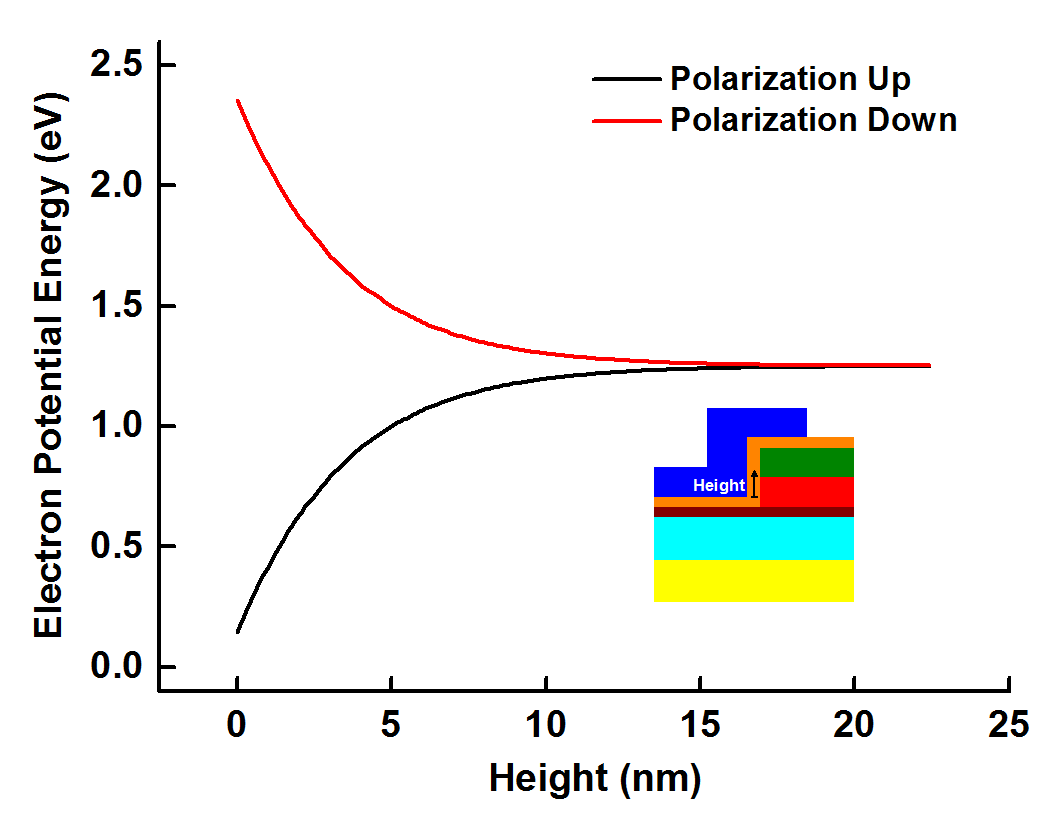


Supplementary Figure 3S.
Energy band profile in the tunnel insulator along the vertical direction for polarization up-state (Black line) and polarization down-state (Red line).

**Supplementary Note 1**

**The variation of the energy band profile of the tunnel insulator**

Supplementary Fig. 1S(a) shows the coordinate system for illustrating the energy band profile of tunnel insulator. As shown in Supplementary Fig. 1S(b) and 1S(c), the energy band profile of tunnel insulator at the bottom (Height=0) changes its shape much by underlying ferroelectric layer. However, far away from the ferroelectric layer (Height=20nm), the energy band profiles of the two opposite polarization directions are very similar to each other. The influence of electric fringe field from the underlying ferroelectric layer on the energy band profile of the tunnel insulator reduces rather quickly as going away from the ferroelectric layer.

**Supplementary Note 2**

**The method for dividing the energy band profile of the tunnel insulator**

Supplementary Fig. 2S represents how to divide the electron energy band profile of the tunnel insulator for calculating the tunnel current density by using mensuration by parts. The calculated potential barrier (black line) is divided into N equal-width rectangular potential barrier segments (orange boxes) and each segment has the potential barrier corresponding to the right edge value of the calculated potential energy. The source electrode is indexed to be j=1, the N rectangular potential barriers to be j=2 to j=N+1, and the drain electrode to be j=N+2.

**Supplementary Note 3**

**The decay of electric fringe field as going away from the ferroelectric layer (Detailed shapes of energy band profiles in the tunnel insulator)**

Supplementary Fig. 3S shows the electron energy band profiles in the tunnel insulator along the vertical direction for both polarization up- and down-state. The height in the horizontal axis is defined from the bottom of tunnel insulator upward as shown in the inset schematic. For polarization up-state, the energy band profile near the bottom of tunnel insulator close to the ferroelectric layer is pulled down and increases as the height increases. In contrast, the energy band profile of polarization down-state is raised near the bottom of tunnel insulator and decreases as the height increases. As can be seen in the figure, the energy band profiles of polarization up- and down-state become quite similar around the height of 15 nm and stay almost the same as the height increases further. This relatively quick decay of electric fringe field is believed to be due to the screening from the source and drain electrodes. Therefore, the large difference of tunnel current between polarization up- and down-state occurs in the relatively small region of tunnel insulator near the underlying ferroelectric layer.
